# Flavonoids and chalcones as selective α-glucosidase inhibitors: from enzymatic screening to validation using the human enzyme

**DOI:** 10.1039/d6md00685j

**Published:** 2026-09-10

**Authors:** Beatriz Vicente, Filipa Amaro, Paula Guedes de Pinho, Carina Proença, M. Luísa Corvo, Eduarda Fernandes, Marisa Freitas

**Affiliations:** a LAQV, REQUIMTE, Laboratory of Applied Chemistry, Department of Chemical Sciences, Faculty of Pharmacy, University of Porto Porto Portugal egracas@ff.up.pt marisafreitas@ff.up.pt; b Associate Laboratory; i4HB-Institute for Health and Bioeconomy, University of Porto 4050-346 Porto Portugal; c UCIBIO-Applied Molecular Biosciences Unit, Laboratory of Toxicology, Faculty of Pharmacy, University of Porto 4050-346 Porto Portugal; d Research Institute for Medicines, Faculdade de Farmácia, Universidade de Lisboa 1649-003 Lisbon Portugal

## Abstract

α-Glucosidase is a key therapeutic target for postprandial glucose control, but most clinically available inhibitors also inhibit α-amylase, leading to gastrointestinal side effects. Natural polyphenols have been widely reported as potent and selective α-glucosidase inhibitors, primarily based on screening assays using yeast enzyme and synthetic substrates. In this study, thirteen flavonoids and five chalcones were initially evaluated using this conventional approach to elucidate the structural determinants of selective α-glucosidase inhibition. Among them, genistein and myricetin emerged as the most potent inhibitors of yeast α-glucosidase, with IC_50_ values significantly lower than that of acarbose, the most widely used α-glucosidase inhibitor. Both flavonoids showed high selectivity, with minimal or no inhibition of α-amylase. To assess the translational relevance of these findings, these compounds were subsequently tested against human α-glucosidase from C2BBe1 cell extracts using the physiological substrate maltose. Under these conditions, only myricetin retained measurable inhibitory activity at low concentrations, which was further validated through chromatographic analysis. These findings demonstrate that current screening strategies relying solely on yeast α-glucosidase can substantially overestimate the inhibitory activity of natural polyphenols. This work highlights the importance of incorporating physiologically relevant enzyme sources, natural substrates, and controls for spectrophotometric assay interference into the discovery of novel α-glucosidase inhibitors, providing a more reliable workflow for the assessment of natural products.

## Abstract 2

The strong α-glucosidase inhibitory activity exhibited by myricetin against both yeast-derived and human enzymes further substantiates the therapeutic potential of polyphenols in diabetes management.

## Graphical abstract

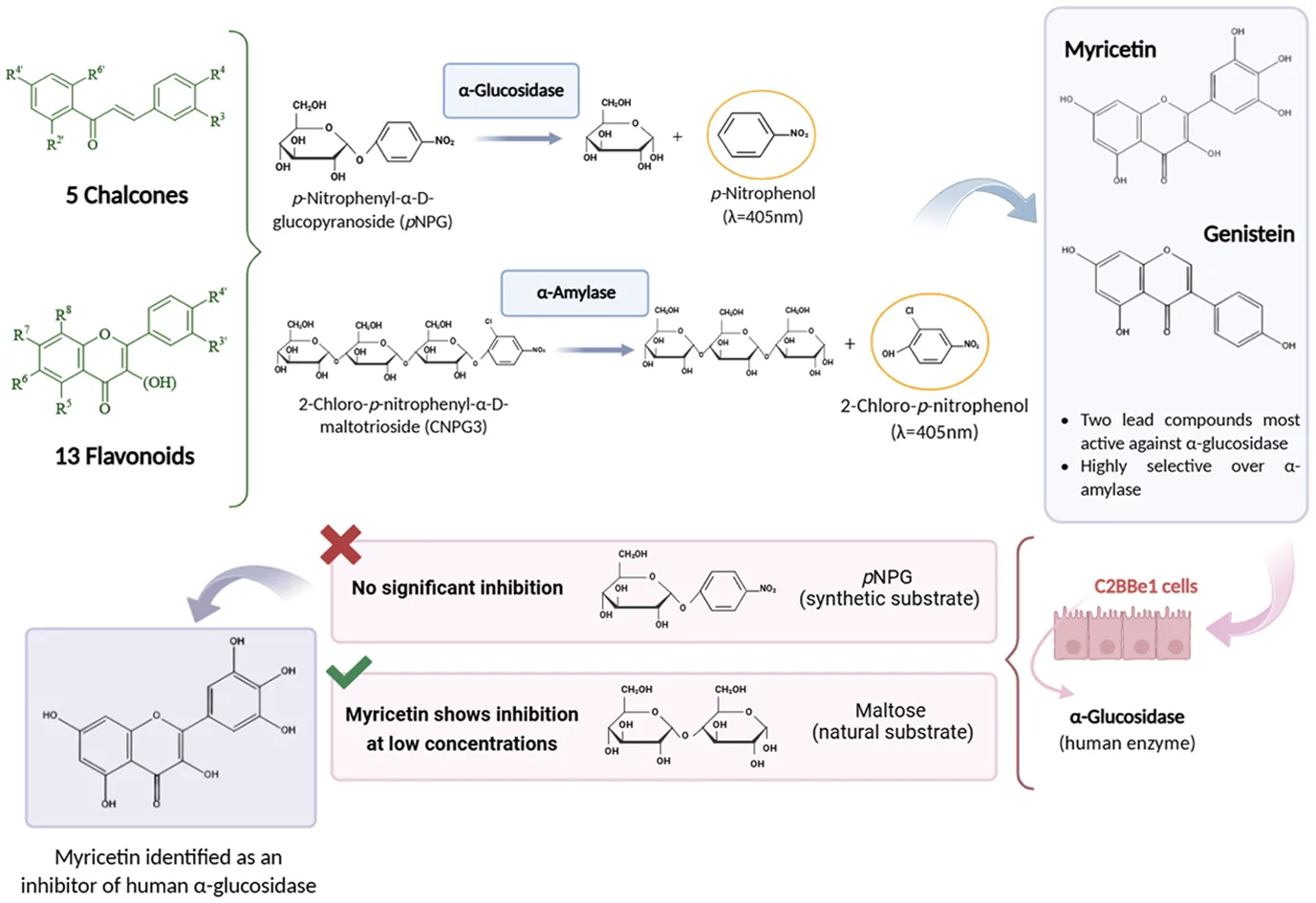

## Introduction

Diabetes mellitus (DM) is one of the main healthcare emergencies, representing one of the ten leading causes of death worldwide.^1,2^ According to the International Diabetes Federation, an estimated 589 million adults between the ages of 20 and 79 years were living with DM in 2024, a number projected to rise by 46% to 853 million by 2050.^3^ Despite remarkable advancements in modern medicine and pharmacological research, these concerning statistics underscore the continuing need to find novel therapeutic approaches.

DM is a chronic endocrine disorder primarily characterised by hyperglycaemia, an abnormal elevation in blood glucose level, resulting from insufficient insulin secretion and/or impaired insulin action.^4–6^ Based on its pathophysiological aetiology, DM is broadly classified into type 1 and type 2, with the latter being the most prevalent form.^7^ Persistent hyperglycaemia is a hallmark pathological feature of both types and can lead to a wide range of acute and chronic complications. Therefore, maintaining optimal blood glucose levels is a primary therapeutic goal in patients with DM.^6,8^

In type 2 DM, glycaemic levels can be regulated by modulating the enzymatic pathways involved in the digestion of dietary glucose sources, such as starch.^6,8^ In the early stages of starch hydrolysis, the polysaccharides are broken down into shorter oligosaccharides and disaccharides (*e.g.*, maltose) through the cleavage of internal α-d-(1→4) glycosidic bonds by α-amylase.^9,10^ Both salivary and pancreatic α-amylases (EC 3.2.1.1) belong to the family of endohydrolases and are classical calcium-containing enzymes.^10,11^ The non-reducing ends of the resulting α-amylolytic products are subsequently hydrolysed by α-glucosidases maltase-glucoamylase (MGAM) and sucrase-isomaltase (SI), each comprising two catalytically active subunits located at the respective C- and N-terminals (Ct and Nt).^5,6,9^ The N-terminal subunit is crucial for the attachment of these enzyme complexes to the apical membrane of small intestinal enterocytes *via* an O-glycosylated stalk domain.^9,12^

All four catalytic subunits of human intestinal α-glucosidases possess high α-1,4-exoglucosidic activity, although each displays distinct substrate specificities. The Ct-MGAM demonstrates maximal activity toward oligosaccharides with up to four glucose residues and is therefore classified as glucoamylase (EC 3.2.1.3), whereas the Nt-MGAM preferentially cleaves disaccharides and is commonly known as maltase (EC 3.2.1.20). Moreover, Ct-SI and Nt-SI subunits display distinctive α-1,2- and α-1,6-glycosidic hydrolysing activities, being referred to as sucrase (EC 3.2.1.48) and isomaltase (EC 3.2.1.10), respectively.^6,12^

In addition to their distinct substrate preferences, α-glucosidases exhibit differential expression throughout the small intestine. For instance, MGAM expression progressively increases along the intestine, reaching its highest level in the distal ileum, whereas SI is predominantly expressed in the jejunum, with lower expression levels observed toward both the proximal and distal regions of the intestine.^9^ While SI constitutes a major component of the apical membrane of human small intestinal epithelial cells, MGAM offsets its comparatively lower abundance by possessing higher hydrolytic activity.^11–13^

At the end of carbohydrate digestion, the monosaccharides (*e.g.*, glucose) generated by glycoside hydrolases are finally absorbed through the apical membrane of enterocytes *via* specific transporters, mainly sodium-glucose co-transporter 1 (SGLT1). Subsequently, their transport across the basolateral membrane into the bloodstream is facilitated by the glucose transporter 2 (GLUT2).^9,14^

Accordingly, inhibition of α-glucosidase can delay starch digestion and subsequent glucose absorption, thereby contributing to the regulation of postprandial glycaemic responses. Despite the availability of clinically approved α-glucosidase inhibitors (acarbose, voglibose, and miglitol), their major limitation lies in their lack of selectivity, as they inhibit both α-amylase and α-glucosidase. This leads to large amounts of undigested carbohydrate reaching the colon, where they undergo microbial fermentation, producing intestinal gas and short-chain fatty acids that ultimately cause various gastrointestinal side effects, including flatulence, bloating, and diarrhoea.^14–16^

To prevent carbohydrate malabsorption and its associated side effects, a more promising strategy is to ensure a slower, yet complete, starch digestion, which has prompted the search for compounds that selectively inhibit α-glucosidase.^14,15^

Over the years, phenolic compounds have garnered increasing attention due to their demonstrated antidiabetic potential.^17^ In addition, polyphenols possess structural features that make them well-suited as enzyme inhibitors, including aromatic rings capable of π–π interactions, as well as several hydroxyl groups that can act as hydrogen bond donors and acceptors.^16^

Flavonoids, the most extensively studied class of polyphenols, are chemically defined by two aromatic rings (A and B rings) linked *via* a three-carbon chain to an oxygenated heterocyclic ring (C ring).^18,19^ Previous studies have reported the selective inhibition of plant-derived flavonoids against starch-digesting enzymes.^14,20,21^ Additionally, as flavonoid precursors, chalcones have received considerable consideration due to their simple structures and wide-ranging pharmacological activities. The term chalcone refers to compounds featuring a 1,3-diarylprop-2-en-1-one framework. Chemically, they are open-chain flavonoids in which two aromatic rings are connected by a three-carbon α, β-unsaturated carbonyl system.^22,23^

In the present study, five chalcones and thirteen structurally related flavonoids (Fig. 1) were selected for the first time to investigate the structural requirements underlying selective α-glucosidase inhibition. Initially, a screening was performed using an enzymatic assay to identify the most promising inhibitors. Then, the inhibitory potential of the selected compounds was evaluated against human α-glucosidase using C2BBe1 cells, a clone of Caco-2 cells derived from human intestinal epithelium, in order to assess their relevance in a human cellular model. Importantly, the novelty of the present work does not lie in the evaluation of new chemical entities, but in establishing a translational strategy for the assessment of α-glucosidase inhibitors. By combining systematic structure–activity relationship (SAR) analysis with validation in a human intestinal enzyme model, this study provides new insights into the physiological relevance of conventional screening assays and in the identification of selective α-glucosidase inhibitors.

**Fig. 1:**
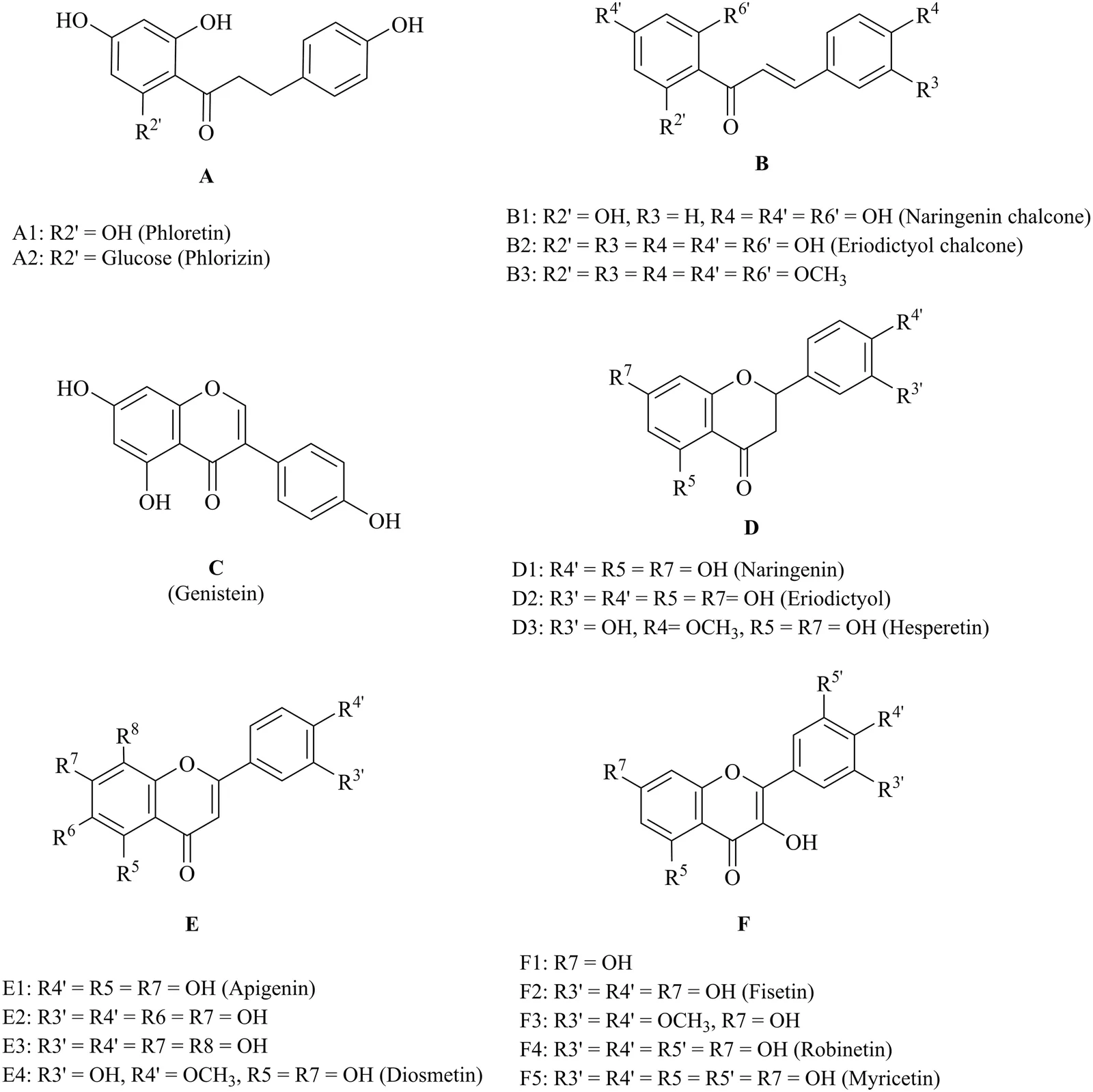
Chemical structures of the studied compounds: (A) dihydrochalcones, (B) chalcones, (C) isoflavone, (D) flavanones, (E) flavones, and (F) flavonols.

## Results

### *In vitro* α-glucosidase inhibition

The inhibitory effects of the studied compounds against α-glucosidase are summarised in Tables 1 and 2. Based on their chemical structures, the studied compounds were divided into six groups: A for dihydrochalcones and B for chalcones (both reported in Table 1), C for isoflavone, D for flavanones, E for flavones, and F for flavonols (all represented in Table 2).

**Table 1:** Inhibitory activity of chalcones against yeast α-glucosidase and pancreatic α-amylase

Chalcones	Structure	R^2′^	R^4′^	R^6′^	R^3^	R^4^	IC_50_ (μM) or inhibition (%) ± SEM	
							α-Glucosidase	α-Amylase
A1 (Phloretin)	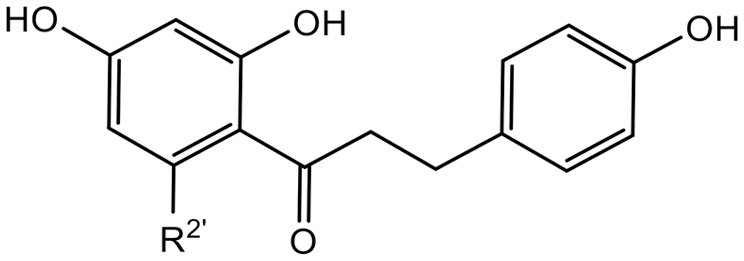	OH	—	—	—	—	68 ± 1 μM	47.1 ± 0.2%^a^^200 μM^
A2 (Phlorizin)		Gluc	—	—	—	—	23 ± 2%^a^^200 μM^	<20%^a^^200 μM^
B1 (Naringenin chalcone)	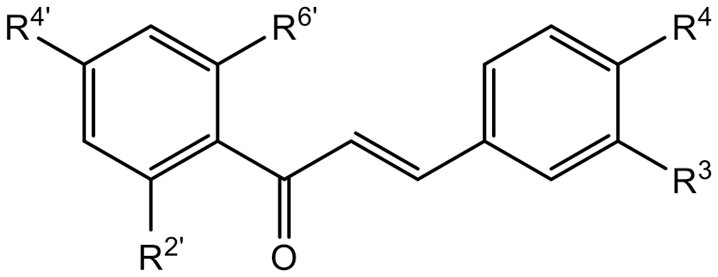	OH	OH	OH	—	OH	31 ± 3%^a^^25 μM^	46 ± 4%^a^^25 μM^
B2 (Eriodictyol chalcone)		OH	OH	OH	OH	OH	43 ± 1%^a^^25 μM^	21 ± 1% μM
B3		OCH_3_	OCH_3_	OCH_3_	OCH_3_	OCH_3_	<20%^a^^100 μM^	<20%^a^^100 μM^

a When IC_50_ could not be determined, the percentage inhibition at the maximum tested concentration is reported. The maximum tested concentrations were established as the highest concentrations that did not absorb at the assay wavelength.

**Table 2:** Inhibitory activity of flavonoids against yeast α-glucosidase and pancreatic α-amylase

Flavonoids	Structure	R^3′^	R^4′^	R^5′^	R^3^	R^5^	R^6^	R^7^	R^8^	IC_50_ (μM) or inhibition (%) ± SEM	
										α-Glucosidase	α-Amylase
C (Genistein)	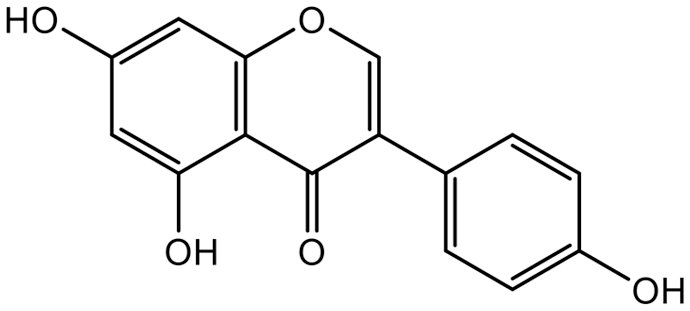	—	—	—	—	—	—	—	—	17 ± 1 μM	153 ± 9 μM
D1 (Naringenin)	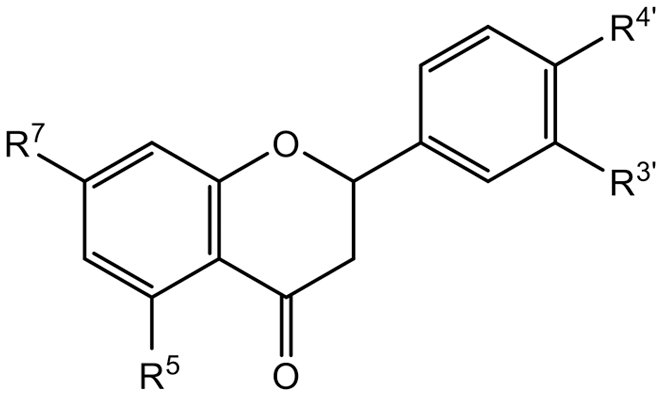	—	OH	—	—	—	—	—	—	137 ± 2 μM	<20%^a^^200 μM^
D2 (Eriodictyol)		OH	OH	—	—	—	—	—	—	47 ± 4%^a^^200 μM^	25 ± 1%^a^^200 μM^
D3 (Hesperetin)		OH	OCH_3_	—	—	—	—	—	—	47 ± 2%^a^^200 μM^	<20%^a^^200 μM^
E1 (Apigenin)	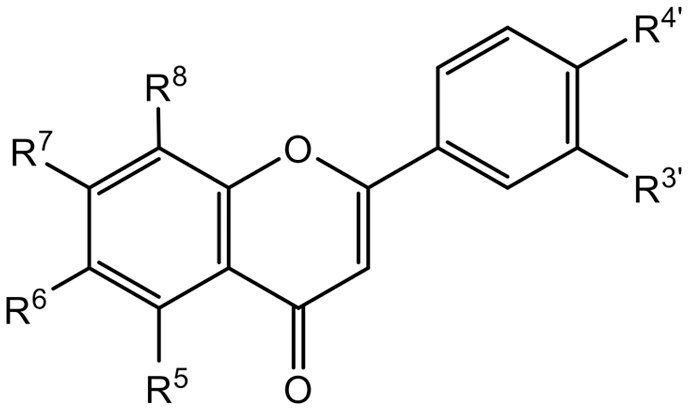	—	OH	OH	—	—	—	OH	—	57 ± 3 μM	60 ± 3 μM
E2		OH	OH	—	—	—	OH	OH	—	41 ± 2%^a^^50 μM^	43 ± 1%^a^^50 μM^
E3		OH	OH	—	—	—	—	OH	OH	31 ± 1 μM	44 ± 2%^a^^50 μM^
E4 (Diosmetin)		OH	OCH_3_	OH	—	—	—	OH	—	<20%^a^^200 μM^	24 ± 2%^a^^100 μM^
F1	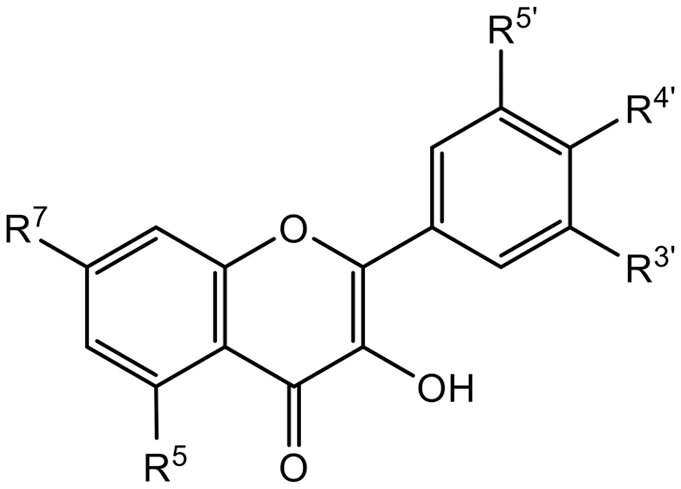	—	—	—	—	—	—	OH	—	<20%^a^^100 μM^	31 ± 1%^a^^50 μM^
F2 (Fisetin)		OH	OH	—	—	—	—	OH	—	40.9 ± 0.3 μM	33 ± 3 μM
F3		OCH_3_	OCH_3_	—	—	—	—	OH	—	<20%^a^^50 μM^	10.0 ± 0.9 μM
F4 (Robinetin)		OH	OH	OH	—	—	—	OH	—	36 ± 2%^a^^50 μM^	29 ± 1 μM
F5 (Myricetin)		OH	OH	OH	—	OH	—	OH	—	14.5 ± 0.6 μM	43 ± 4%^a^^25 μM^
**Positive control:** acarbose	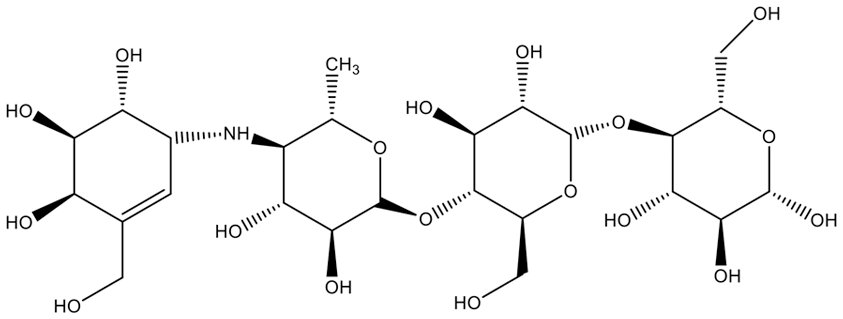	—	—	—	—	—	—	—	—	267 ± 12 μM	0.88 ± 0.03 μM

a When IC_50_ could not be determined, the percentage inhibition at the maximum tested concentration is reported. The maximum tested concentrations were established as the highest concentrations that did not absorb at the assay wavelength.

As shown in Table 1, only the dihydrochalcone phloretin (A1) exhibited inhibitory activity against α-glucosidase. To determine if the addition of a sugar moiety, more specifically glucose, could enhance the activity of A1, its glycosylated derivative, the compound phlorizin (A2), was also investigated. However, this latter did not display inhibitory activity at the highest concentration tested (200 μM), indicating that glycosylation at position 2′ leads to a loss of activity. Notably, among the chalcone-related compounds, only the dihydrochalcone A1 showed some inhibitory activity. In contrast, its corresponding chalcone, naringenin chalcone (B1) did not reach 50% inhibition at the highest concentration tested (25 μM), thus precluding IC_50_ determination. Nevertheless, these data do not allow us to draw a definitive conclusion regarding the role of the α-β double bond in inhibitory activity, as A1 exhibited an IC_50_ of 68 ± 1 μM, whereas B1 could not be evaluated at concentrations exceeding 25 μM.

Regarding the flavonoid group, six of the thirteen compounds tested demonstrated activity against α-glucosidase: the isoflavone (C), one flavanone (D1), two flavones (E1 and E3) and two flavonols (F2 and F5). Among these, the flavanone naringenin (D1) was the least active, with an IC_50_ of 137 ± 2 μM. Analysing the flavanone series (D1–3), we can conclude that the introduction of a catechol group on the B ring is unfavorable for the inhibitory activity. Indeed, eriodicytol (D2), which differs from D1 by the presence of an additional hydroxyl group at position 3′ of the B ring, achieved 50% inhibition of α-glucosidase only at concentrations close to 200 μM. In contrast, the methoxylation at position 4′ of the B ring appears to have minimal impact on activity, as evidenced by the similar inhibition percentages observed at 200 μM for D2 and hesperetin (D3).

Myricetin (F5) and the isoflavone genistein (C) emerged as the most potent α-glucosidase inhibitors, presenting an IC_50_ of 14.5 ± 0.6 μM and 17 ± 1 μM, respectively. Notably, both share the presence of hydroxyl groups at positions 5 and 7 of the A ring, underscoring the importance of these substituents at these same positions for α-glucosidase inhibitory activity.

An analysis focused on the most active compound, myricetin (F5), and comparing it with the other flavonols (F1–4) tested allows us to make SAR considerations.

Comparison of F5 with fisetin (F2) highlights the importance of hydroxyl groups at positions 5′ and 5, as their absence results in an almost threefold increase in the concentration required to inhibit 50% of α-glucosidase activity. Furthermore, the relevance of the catechol moiety of the B ring within the flavanol series was supported by the lack of significant activity observed for compound F1 at concentrations up to 100 μM, whereas F2 exhibited an IC_50_ value of 40.9 ± 0.3 μM. Finally, methoxylation at positions 3′ and 4′ appears to be disadvantageous, as can be inferred from comparing F2 with its 3′,4′-dimethylated derivative, fisetin-3′,4′-dimethylether (F3), which showed less than 20% inhibition at 50 μM.

### *In vitro* pancreatic α-amylase inhibition

Based on the results presented in Table 1, only eriodictyol chalcone (B2) demonstrated significant inhibitory activity against pancreatic α-amylase, positioning it as the second most potent compound after fisetin-3,4′-dimethylether (F3). Notably, comparing F3 with fisetin (F2), we can conclude that the double methylation of the B ring enhances inhibitory activity against α-amylase, as F3 presented an IC_50_ that is over threefold lower than F2. Furthermore, comparison of F2 with 7-hydroxyflavonol (F1) emphasizes the contribution of the catechol group, and comparison with robinetin (F4) suggests that the presence of an additional hydroxyl group at position 5 of the B ring further increases α-amylase inhibition.

However, none of the compounds tested surpassed the positive control, acarbose, in α-amylase inhibition. In contrast, for α-glucosidase, all seven active compounds exhibited IC_50_ values lower than that of acarbose, demonstrating their superior inhibitory capacity.

Among the most potent α-glucosidase inhibitors, myricetin (F5) had no effect on α-amylase activity at the highest concentration tested (25 μM), yet inhibited α-glucosidase with an IC_50_ of 14.5 ± 0.6 μM. On the other hand, genistein (C) showed modest α-amylase inhibition, since the concentration required to achieve 50% inhibition was nearly nine times higher than that needed for α-glucosidase, highlighting its relative selectivity.

### Mechanism of α-glucosidase inhibition

Based on the results described above, the most potent and selective α-glucosidase inhibitors, along with the positive control, acarbose, were selected for subsequent determination of their inhibition mechanisms, as deduced from statistical analysis of the experimental data fitted to the Michaelis–Menten kinetic model and corresponding Lineweaver–Burk plots.

As depicted in Fig. 2, both flavonoids presented a mixed-type inhibition mechanism, as increasing compound concentrations resulted in decreased *V*_max_ values, while *K*_m_ value can either decrease or increase. On the other hand, and consistent with earlier reports, acarbose exhibited a competitive mode of inhibition, characterised by an increase in *K*_m_ value with rising inhibitor concentrations, while *V*_max_ remained unchanged. In mixed-type inhibition, the inhibitor can bind either to the enzyme's active site or to an allosteric site, whereas in the competitive mechanism, the inhibitor can only interact with the enzyme in the active site.^24,25^

**Fig. 2:**
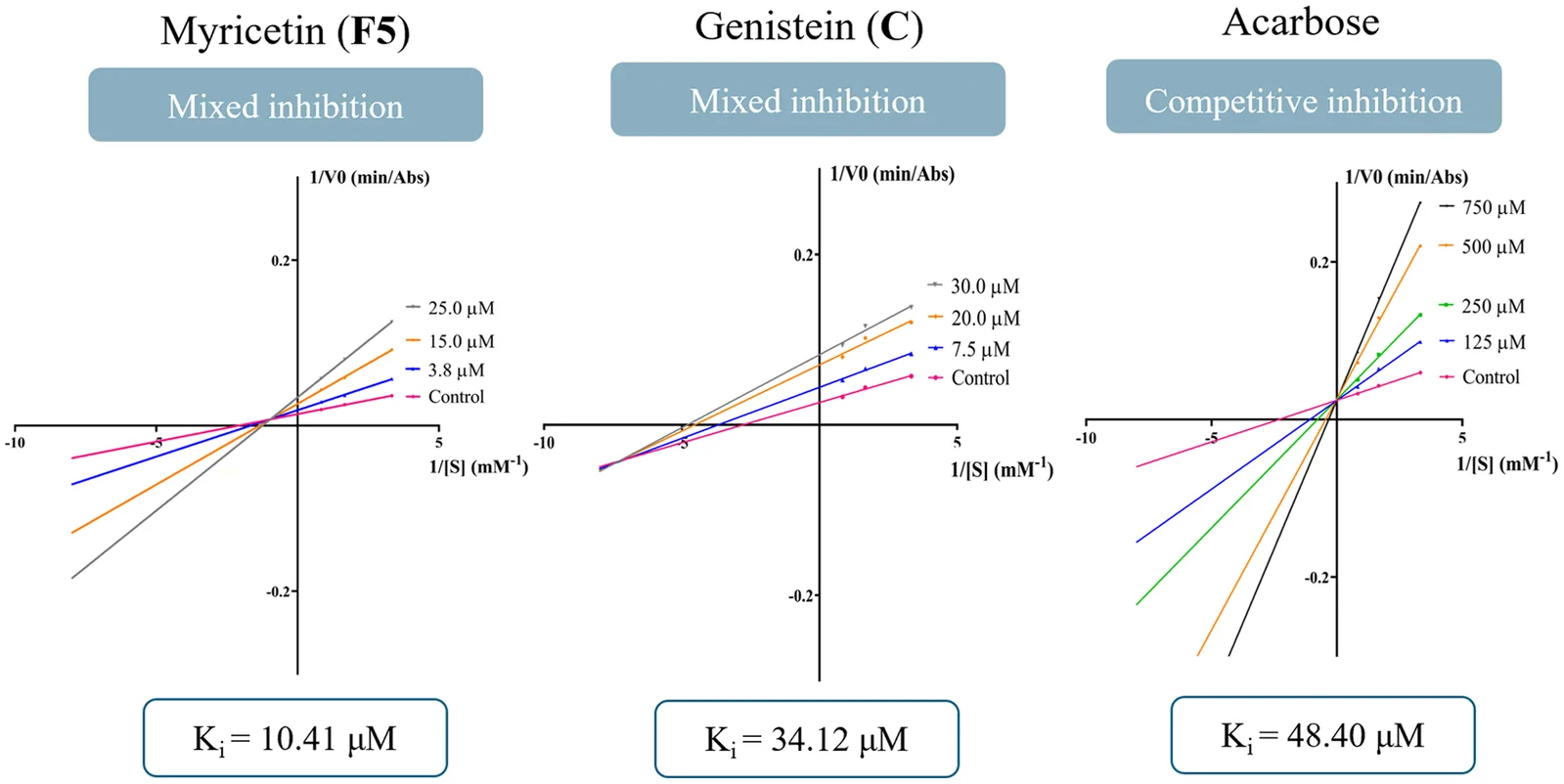
Lineweaver–Burk plots of α-glucosidase inhibition by myricetin, genistein, and acarbose, the positive control.

### Cell viability: MTT assay

The impact of the two most effective and selective α-glucosidase inhibitors, along with the positive control, acarbose, on C2BBe1 cell viability was assessed using the MTT colorimetric assay after 10 minutes and 24 hours of incubation. As shown in Fig. 3, none of the tested concentrations induced a statistically significant reduction in cell viability compared with untreated cells. Based on these findings, 50 μM for the flavonoids and 200 μM for acarbose were selected as the maximum non-cytotoxic concentrations for the following experiments. Triton X-100 (1%) was used as a positive control, reducing cell viability to less than 20% and thereby confirming the reliability of the protocol.

**Fig. 3:**
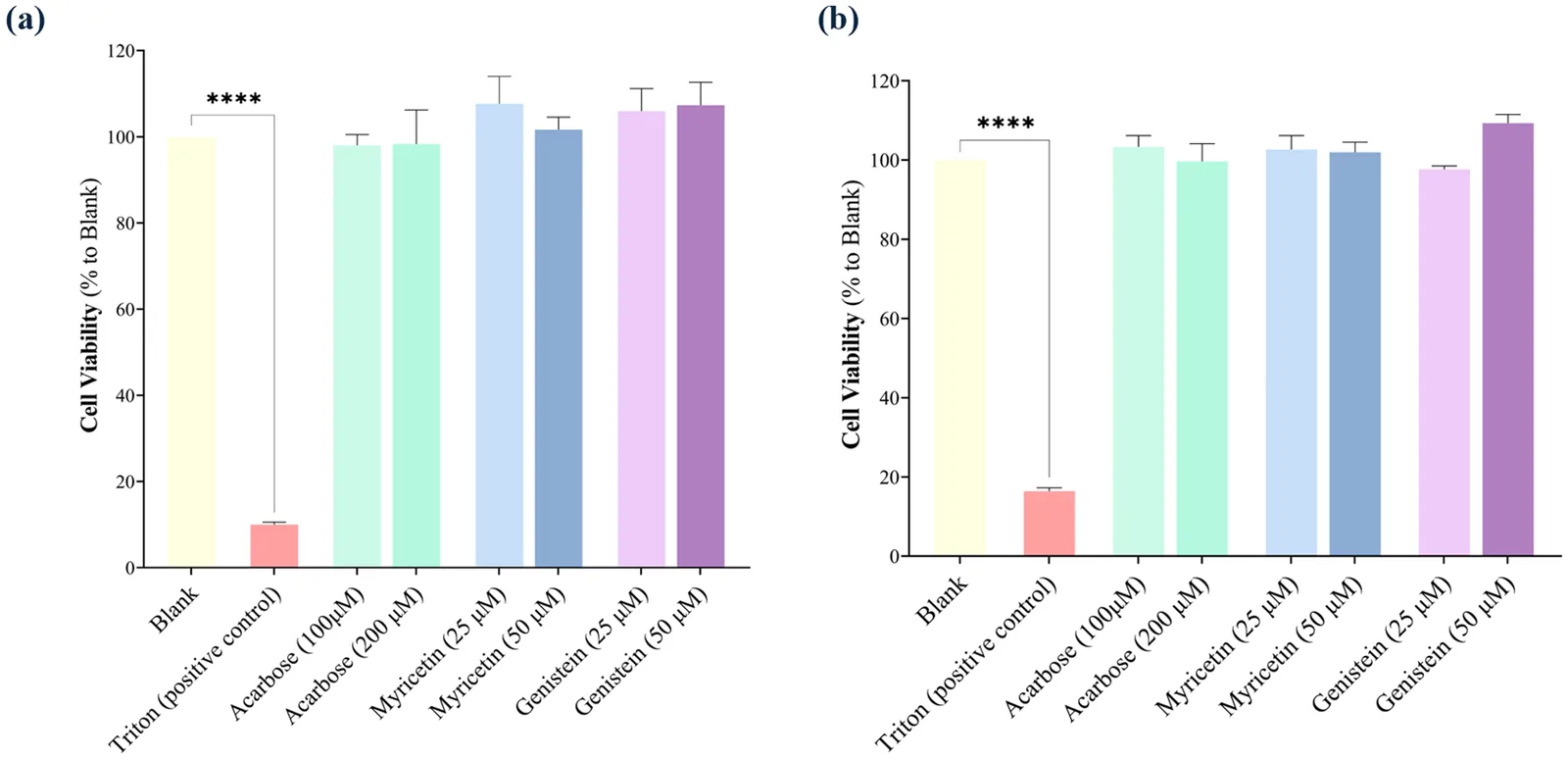
Assessment of cell viability by MTT assay following 10 minutes (a) and 24 hours (b) of exposure. Statistical significance indicated by **** *p* < 0.0001, compared to untreated cells.

### Sucrase-isomaltase inhibitory activity using *p*NPG as the substrate

To enable a direct comparison of the inhibitory activity of the tested compounds using two different enzyme sources, the substrate *p*-nitrophenyl-α-d-glucopyranoside (*p*NPG) used in the yeast α-glucosidase assay, was also employed in the assay with human enzyme. The highest concentration tested for each flavonoid, selected based on the results of the MTT assay, as well as the maximum concentration used for the positive control, did not interfere with the assay. Even though both myricetin and genistein exhibited greater inhibitory activity than the positive control in the yeast enzyme assay, neither compound showed significant activity against human SI at the highest concentration tested, whereas acarbose presented an IC_50_ value of 91 ± 3 μM (Table 3).

**Table 3:** Inhibitory activity of the selected flavonoids and positive control against human α-glucosidase activity in C2BBe1 cell extracts using *p*NPG or maltose as substrates

Flavonoids	Structure	IC_50_ (μM) or inhibition (%) ± SEM	
		*p*NPG	Maltose
Myricetin	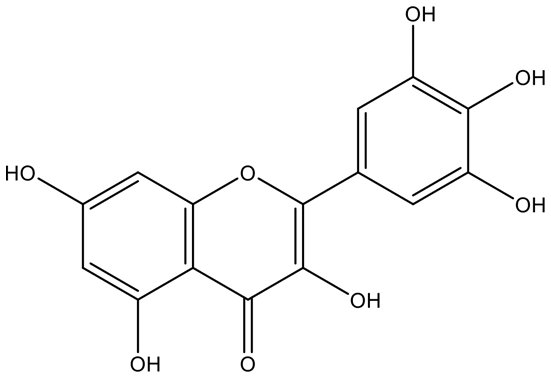	27.6 ± 0.6%^a^^50 μM^	27 ± 3%^b^^6.25 μM^
Genistein	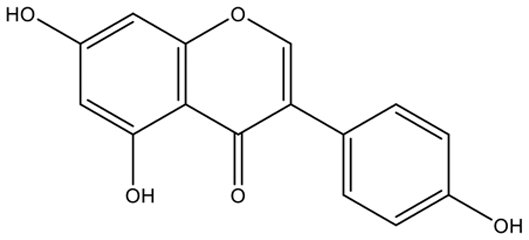	<10%^a^^50 μM^	<15%^b^^50 μM^
Positive control: acarbose	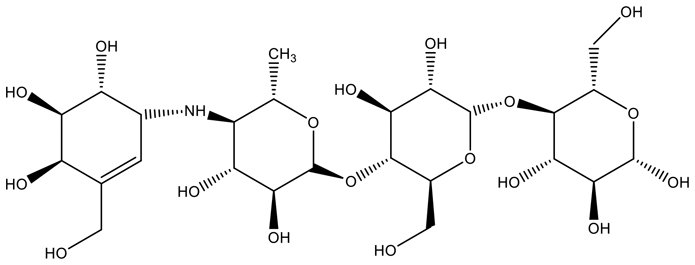	91 ± 3 μM	11 ± 4 μM

a The maximum tested concentrations were established as the highest concentrations that did not absorb at the assay wavelength.

b The maximum tested concentrations were defined as the highest concentrations that did not interfere with the colorimetric glucose oxidase assay.

### Maltase inhibitory activity – glucose oxidase assay

To investigate the inhibitory effects of the selected flavonoids on human α-glucosidase using both synthetic and physiological substrates, enzyme activity was further evaluated using maltose as a natural substrate. For this purpose, a glucose oxidase-peroxidase-based assay was established. Initially, the potential interference of the tested compounds with the assay kit was assessed, and only concentrations that caused less than 20% inhibition of the glucose oxidase-peroxidase developing reaction were selected for further experiments.

Consistent with previous findings, none of the selected flavonoids achieved 50% inhibition of the enzyme (Table 3). However, myricetin exhibited moderate inhibitory activity, reducing enzyme activity by 27 ± 3% at 6.25 μM.

Nevertheless, this flavonol interfered with the assay reaction, either by directly interacting with the enzymatic kit components or by scavenging hydrogen peroxide generated during the reaction. Since hydrogen peroxide is required for the formation of the quinoneimine chromophore, whose absorbance is directly proportional to glucose concentration, this interference may affect the accuracy of the measured inhibitory effect.

Based on these observations, further evaluation of myricetin at concentrations above 6.25 μM would be of interest.

To overcome the limitations associated with the enzymatic determination of glucose, chromatographic detection by gas chromatography–mass spectrometry (GC–MS) was investigated as an alternative approach.

### GC–MS

As previously mentioned, myricetin was found to interfere with the glucose oxidase assay. Therefore, GC–MS analysis of the reaction product (glucose) was assessed as an alternative to enzymatic detection. Inhibition was readily observed as a decrease in the glucose peak areas (semiquantitative analysis) (Fig. 4), whereas the substrate peaks (maltose) were not routinely quantified due to their saturating concentration. This approach allowed us to test higher concentrations of myricetin, making it possible to achieve inhibition levels greater than 50%. At 25 μM, myricetin inhibited enzyme activity by 59 ± 2%. These findings demonstrate that myricetin is active against human α-glucosidase. In contrast, acarbose exhibited a comparable inhibitory effect (63 ± 4% of inhibition) at a lower concentration, 12.5 μM.

**Fig. 4:**
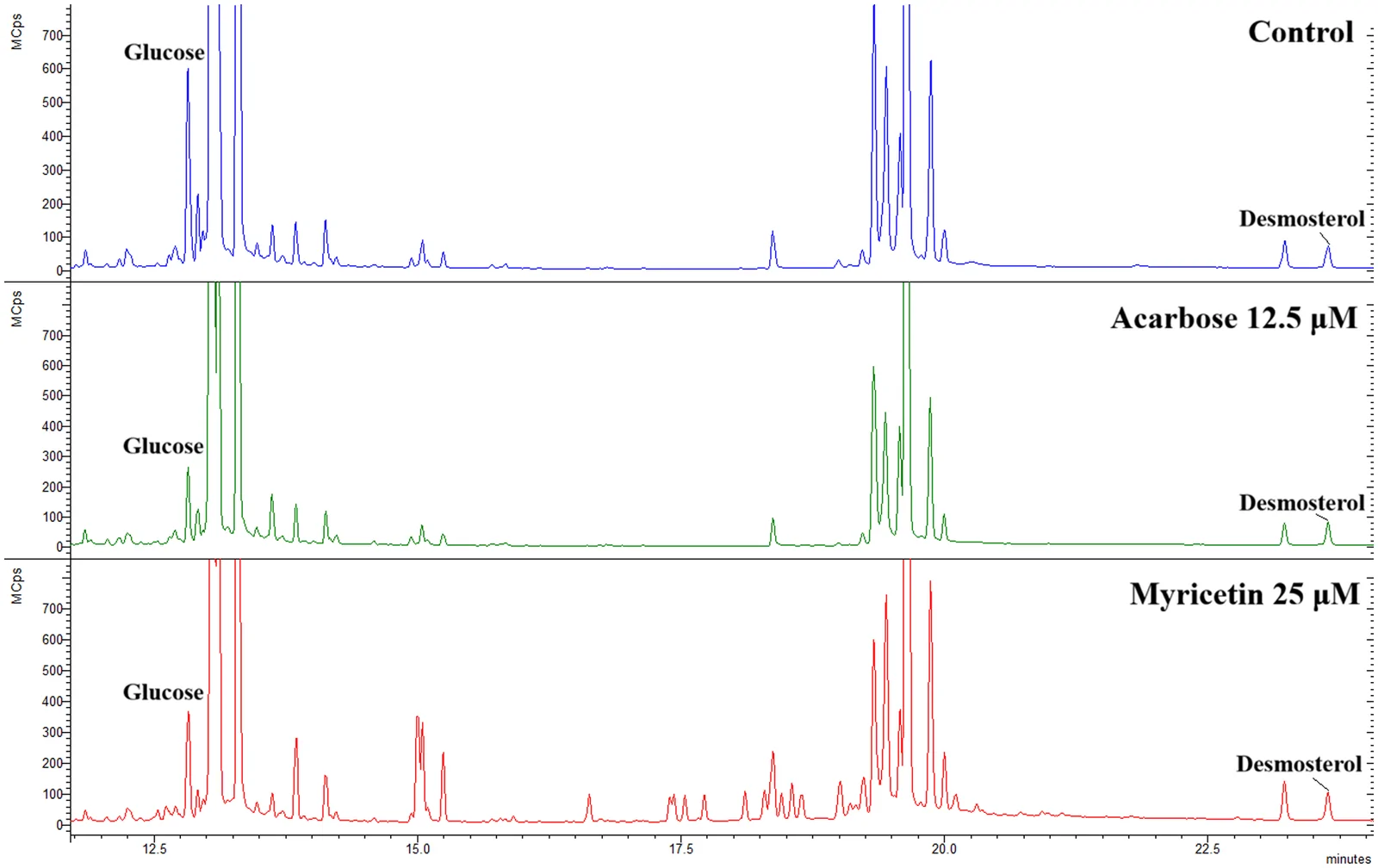
Representative GC–MS chromatograms derived from maltase assays conducted in the absence (control) and in the presence of inhibitors, acarbose (12.5 μM) or myricetin (25 μM). The glucose peak area at retention time of 12.82 minutes was selected for integration and normalised to the peak area of desmosterol (internal standard).

## Discussion

Among the evaluated compounds in the present study, only four exhibited selective inhibitory activity against α-glucosidase at the highest concentration tested: the dihydrochalcone phloretin (A1) and the flavonoids naringenin (D1), 7,8,3′,4′-tetrahydroxyflavone (E3), and myricetin (F5). Additionally, two other flavonoids – apigenin (E1) and fisetin (F2) – also inhibited α-glucosidase; however, their IC_50_ values were comparable to those observed for α-amylase, indicating a lack of selectivity. Notably, genistein (C) also emerges as a promising candidate, with the second-lowest IC_50_ value against α-glucosidase (17 ± 1 μM), surpassed only by myricetin (14.5 ± 0.6 μM). Moreover, substantially higher concentrations of genistein were required to achieve 50% inhibition of α-amylase (153 ± 9 μM), suggesting a preferential inhibitory effect toward α-glucosidase. Collectively, the obtained results suggest that flavonoids could be promising α-glucosidase inhibitors with fewer gastrointestinal side effects.

Focusing on the structural features favouring α-glucosidase inhibition, hydroxylation of flavonoids appears to enhance their inhibitory potency, whereas methoxylation and glycosylation tend to reduce enzymatic inhibition.^17^ This trend is illustrated by comparing fisetin-3,4′-dimethylether (F3) with fisetin (F2), and phlorizin (A2) with phloretin (A1), respectively. The disadvantageous effect of methoxylation is more pronounced when –OH groups at positions 3′ and 4′ of the B ring are replaced by –OCH_3_ groups. As observed in F3 and F2, the substitution of both positions resulted in activity loss: at a concentration of 50 μM, F3 inhibited less than 20% of α-glucosidase activity, whereas F2 exhibited an IC_50_ of 40.9 ± 0.3 μM.^26^ Meanwhile, comparison of hesperetin (D3) with eriodictyol (D2) indicates that methoxylation at position 4′ of the B ring has a less pronounced impact, as both compounds inhibited approximately 47% of enzyme activity at 200 μM.

In agreement with Proença *et al.*,^20,26^ the presence of an –OH group at position 5 of the A ring appears to be critical for the inhibitory activity of flavonoids against α-glucosidase. Notably, myricetin (F5) was the most potent inhibitor, exhibiting the lowest IC_50_ value (14.5 ± 0.6 μM), while its structural analogue, robinetin (F4), which lacks the hydroxyl group at position 5, inhibited only 36% of enzyme activity at 50 μM. Moreover, the relevance of hydroxyl groups at position 8 of the A ring was also confirmed, as shifting the –OH group from position 8 (E3) to position 6 (E2) led to a loss of activity. Additionally, the presence of a catechol moiety in the flavonol scaffold appears to be advantageous, as the existence of this group in F2 is associated with markedly higher inhibitory activity compared with 7-hydroxyflavonol (F1), which was inactive at the highest concentration tested (100 μM).

Regarding α-amylase inhibition, Sales *et al.*^10^ reported that interactions between polyphenols and the catalytic site of this enzyme involve hydrogen bond formation with hydroxyl groups at 4′ and 5′ of the B ring. This observation was further supported by Guo *et al.*,^15^ and it is consistent with the present findings, as F2 displayed an IC_50_ value of 33 ± 3 μM whereas F4, with an additional –OH group at position 5′, presented a lower IC_50_ value (29 ± 1 μM), indicating enhanced inhibitory potency. The importance of the hydroxyl group at position 4′ is also evident, as methoxylation at this position reduced the inhibitory activity: D2 at 200 μM inhibited 25% of enzyme activity, whereas its methoxylated analogue (D3) inhibited less than 20% at the same concentration. Furthermore, the presence of a catechol group at positions 3′ and 4′ proved to be beneficial, as while compound F1 inhibited only 31% of enzyme activity at 50 μM, compound F2, bearing this additional group, achieved 50% of inhibition at a concentration of 33 ± 3 μM. Interestingly, methoxylation of both –OH groups further enhanced the inhibitory activity, as the dimethoxylated derivative F3 exhibited an IC_50_ value of 10.0 ± 0.9 μM, being inclusively the most active compound against α-amylase within this panel. Conversely, and in agreement with Proença *et al.*,^27^ glycosylation of the flavonoid scaffold did not favour α-amylase inhibition, possibly due to increased molecular size and polarity. This was evidenced by comparing A1 and A2: while the first inhibited 47% of enzyme activity at 200 μM, its glycosylated counterpart was essentially inactive (less than 20% inhibition) at the same concentration. Finally, in the case of dihydrochalcones, a subclass of chalcones in which the double bond of the three-carbon bridge is reduced,^23^ the absence of this double bond may be disadvantageous. Although naringenin chalcone (B1) and A1 demonstrated similar percentages of inhibition, A1 required an eight-fold higher concentration to achieve a comparable inhibitory effect. These findings may suggest a contribution of the α, β-unsaturated system to α-amylase inhibition; however, a definitive conclusion cannot be drawn because B1 could not be evaluated at concentrations above 25 μM.

These structural requirements reflect fundamental differences in the protein structures of α-amylase and α-glucosidase. As an endo-acting enzyme, the catalytically active site of α-amylase is located within a wide and shallow groove on the protein surface, whereas α-glucosidase, as an exo-acting enzyme, displays a narrow and deep catalytic pocket.^14^

Overall, myricetin (F5) showed the strongest *in vitro* inhibitory effect against α-glucosidase, with an IC_50_ value of 14.5 ± 0.6 μM, closely aligning with the value recorded by Jia *et al.*: 11.63 ± 0.36 μM.^28^ In contrast, this flavonol had no inhibitory effect against α-amylase at the highest concentration tested (25 μM).

In agreement with our findings, Kang *et al.*^29^ previously demonstrated the α-glucosidase inhibitory effect of myricetin *in vitro*, using rat intestinal maltase as the enzyme source and either *p*NPG or maltose as substrates. In addition to reducing small intestinal maltase activity, myricetin administration significantly decreased fasting serum glucose levels and blood glycated haemoglobin *in vivo*. These findings led the authors to conclude that myricetin effectively controls both fasting and postprandial hyperglycaemia in diabetic animal models.

Furthermore, genistein emerged as the second most potent α-glucosidase inhibitor. Although this isoflavone was also capable of inhibiting α-amylase, it displayed an IC_50_ value for α-amylase that was nearly nine-fold higher than that observed for α-glucosidase, indicating greater selectivity toward the latter, consistent with the findings of Jia *et al.*^28^

Our results align with earlier studies that described myricetin and genistein as mixed-type inhibitors of α-glucosidase. This indicates that both compounds can bind not only to the active site of the enzyme, thereby competing with the substrate, but also to allosteric sites, interacting with functional groups outside the catalytic centre.^24,25^

As in most previous studies, the compounds under study were initially tested against yeast α-glucosidase. However, this model provides limited predictive value for human α-glucosidase inhibition since the yeast enzyme typically belongs to the GH13 glycoside hydrolase family, whereas the human enzyme is a member of the GH31 family. Although both GH13 and GH31 α-glucosidases catalyse the hydrolysis of α-glycosidic bonds and share the characteristic (β/α)_8_-barrel fold in their catalytic domain, they differ considerably in their overall structure, with GH31 enzymes being composed of five structural domains, whereas GH13 presents only three domains.^30,31^ These structural differences extend to their active-site pockets, influencing both binding affinity and inhibitory mechanism. GH13 α-glucosidases generally possess a narrower, pocket-shaped active site, whereas GH31 enzymes feature a larger and more accessible catalytic pocket.^30^ Consequently, inhibitory activity observed against yeast α-glucosidase may not accurately reflect inhibition of the human enzyme. To address this shortcoming, an inhibition assay was optimised using human C2BBe1 cell-free extracts as the enzyme source. Compared with the parental Caco-2 cell line, C2BBe1 cells exhibit a more homogeneous brush border and express intestinal enzymes at levels closer to those observed in human enterocytes.^32–34^ In particular, these cells display increased expression of brush-border enzymes such as sucrase–isomaltase during differentiation, making them a suitable model to study intestinal α-glucosidase activity and to evaluate the potential physiological relevance of the tested compounds.^32,35^

This assay was employed to assess the inhibitory activity of the two most promising α-glucosidase inhibitors, and the results were compared with those obtained using the yeast enzyme. Notably, in the *p*NPG assay, both myricetin and genistein were ineffective against the human enzyme at the highest concentration tested (50 μM). However, since this assay employs a synthetic substrate that may not accurately reflect the natural enzymatic process, the glucose oxidase assay can serve as an alternative method to quantify the amount of glucose generated from the hydrolysis of natural substrates by α-glucosidase. Nonetheless, this assay is susceptible to interference, particularly from reducing compounds such as polyphenols. The radical scavenging property of flavonoids is largely determined by the number and position of –OH groups within their structure. As demonstrated by Wong *et al.*,^36^ compounds with multiple –OH groups, especially in the ortho position of the B ring, can cause substantial interference. Indeed, this was observed for myricetin, for which 6.25 μM was the highest concentration that could be tested while maintaining assay interference below 20%. Notably, at this concentration, this flavonol inhibited 27 ± 3% of human α-glucosidase activity as determined by the glucose oxidase assay. To assess higher concentrations and achieve greater analytical sensitivity without interference, GC–MS was subsequently employed. Using this method, inhibition above 50% was observed at a concentration of 25 μM, thereby demonstrating the inhibitory potential of myricetin against human α-glucosidase, albeit with lower potency than acarbose. Nevertheless, myricetin retained greater selectivity for α-glucosidase over α-amylase, a pharmacological property that may contribute to reducing the gastrointestinal adverse effects commonly associated with current α-glucosidase inhibitors. More importantly, the discrepancy observed between yeast and human α-glucosidase inhibition demonstrates that conventional screening assays may overestimate the translational potential of candidate inhibitors.

In accordance with these findings, this study not only extends the SAR analysis to chalcones and validates the most promising compounds in a human intestinal model but also introduces a GC–MS-based method that overcomes the analytical limitations of conventional glucose oxidase and spectrophotometric assays when evaluating polyphenolic compounds. Collectively, these advances establish a more reliable and translational workflow for the identification and validation of selective α-glucosidase inhibitors.

## Experimental

### Chemicals

The following reagents were purchased from Sigma-Aldrich Co. (St. Louis, MO, USA): α-glucosidase from *Saccharomyces cerevisiae*, α-amylase from porcine pancreas, *p*-nitrophenyl-α-d-glucopyranoside (*p*NPG), 2-chloro-*p*-nitrophenyl-α-d-maltotrioside (CNPG3), acarbose, dimethyl sulfoxide (DMSO), sodium hydrogen phosphate (Na_2_HPO_4_), Dulbecco's phosphate-buffered saline (DPBS) without calcium chloride and magnesium chloride, 0.4% trypan blue solution, Triton X-100 (TX), 3-(4,5-dimethylthiazol-2-yl)-2,5-diphenyl-tetrazolium bromide (MTT), protease inhibitor cocktail (Mini Complete, Roche), bovine serum albumin (BSA), glucose, maltose, glucose assay kit MAK476, chloroform, desmosterol, methoxyamine hydrochloride (MOX), *N*,*O*-bis-(trimethylsilyl) trifluoroacetamide with 1% of trimethylchlorosilane (BSTFA + TMCS), chalcone A1 (phloretin), flavonoids D1 (naringenin), D3 (hesperetin), E1 (apigenin) and E4 (diosmetin). Compounds B1 (naringenin chalcone), B2 (eriodictyol chalcone), D2 (eriodictyol), F1 (7-hydroxyflavonol), F4 (robinetin) and F5 (myricetin) were obtained from Extrasynthese (Lyon Nord, France). Flavonoids E2 (6,7,3′,4′- tetrahydroxyflavone) and F2 (fisetin) were obtained from Biosynth (Bratislava, SK). B3 (3,4,2′,4′,6′-pentamethoxychalcone), E3 (7,8,3′,4′-tetrahydroxyflavone) and F3 (fisetin-3,4′-dimethylether) were purchased from Indofine Chemical Company, Inc. (Hillsborough, NJ, USA). The remaining compounds, A2 (phlorizin) and C (genistein) were obtained from Tocris Bioscience (Bristol, UK) and Santa Cruz Biotechnology (Dallas, TX, USA), respectively. All compounds tested exhibited a purity of ≥95%, according to their respective commercial suppliers.

Dulbecco's modified eagle's medium (DMEM) high glucose was obtained from Avantor/VWR (Radnor, PA, USA). DMEM (high glucose, no glutamine, no phenol red) and trypsin-(ethylenediaminetetraacetic acid) EDTA 0.25% were purchased from ThermoFisher Scientific (Waltham, MA, USA).

Foetal bovine serum (FBS) and the penicillin–streptomycin solution were obtained from Corning© (New York, USA). Human transferrin was purchased from PAN-Biotech GmBH (Aidenbach, Germany).

Protein Assay Reagents A, B and S were obtained from Bio-Rad Laboratories (Hercules, CA, USA).

The human colorectal adenocarcinoma cell line C2BBe1 (ATCC© CRL-2102™) was purchased from the American Type Culture Collection and cultured according to their specific instructions.

### *In vitro* α-glucosidase inhibition assay

α-Glucosidase activity was measured using a method previously reported by Rocha *et al.*^37^ The assay was performed by monitoring the α-glucosidase-mediated transformation of the substrate *p*NPG into α-d-glucose and *p*-nitrophenol, at 405 nm. Briefly, in a 96-well plate, the enzyme (0.04 U mL^−1^) dissolved in 100 mM phosphate buffer (pH 6.8) was pre-incubated at 37 °C for 5 minutes with the compounds under study dissolved in DMSO. The catalytic reaction was initiated by adding *p*NPG (600 μM) dissolved in phosphate buffer, and the reaction mixture was incubated at 37 °C for 30 minutes. All concentrations refer to the final volume of the reaction mixture. The enzymatic reaction was monitored spectrophotometrically for 30 minutes at 37 °C in a microplate reader (Synergy HT, Bio-Tek) with the wavelength set to 405 nm. To determine the % inhibition of α-glucosidase activity, the values of the absorbance *versus* time plots in the interval of 5 to 15 minutes were obtained and considered for further calculation. Acarbose (0–3000 μM) was used as a positive control.

### *In vitro* pancreatic α-amylase inhibition assay

The α-amylase activity was measured using a method previously reported by Rocha *et al.*^37^ The α-amylase-mediated hydrolysis of the substrate CNPG3 into 2-chloro-nitrophenol (CNP), 2-chloro-4-nitrophenyl-α-d-maltoside (CNPG2), maltotriose and glucose was monitored. In brief, in a 96-well plate, the enzyme (0.1 U mL^−1^) dissolved in 20 mM phosphate buffer (pH 6.8) was exposed to the compounds under study dissolved in DMSO. After incubation for 10 minutes at 37 °C, the substrate CNPG3 (500 μM) dissolved in phosphate buffer was added. All concentrations refer to the final volume of the reaction mixture. The reaction was monitored for 30 minutes at 37 °C in a microplate reader (Synergy HT, BIO-TEK), with the wavelength set to 405 nm. To determine the % inhibition of α-amylase activity, the derivative values of the absorbance *versus* time plots in the interval of 5 to 15 minutes were obtained and considered for further calculation. Acarbose (0–20 μM) was used as a positive control.

### Study of the α-glucosidase inhibition type

The types of α-glucosidase inhibition were determined for the most active compounds, genistein (0–30 μM), myricetin (0–25 μM), and the positive control, acarbose (0–750 μM). In a 96-well plate, 0.04 U mL^−1^ α-glucosidase, dissolved in 100 mM phosphate buffer (pH 6.8), was pre-incubated with the previously mentioned compounds for 5 minutes at 37 °C. Then, the substrate *p*NPG was added at three different concentrations (300, 600, and 1200 μM), and the reaction mixture was incubated at 37 °C for 30 minutes in a microplate reader (Synergy HT, BIO-TEK), with the wavelength set to 405 nm. Then, the values of the absorbance *versus* time plots in the interval of 5 to 15 minutes were obtained and used for further processing. The study of the inhibition type was performed using the non-linear regression Michaelis–Menten enzyme kinetics and the corresponding Lineweaver–Burk double reciprocal plots for each concentration of the inhibitor and substrate. The results presented correspond to at least three independent experiments.

### Cell culture

C2BBe1 were seeded in 75 cm^2^ culture flask in DMEM medium supplemented with 0.01 mg ml^−1^ human transferrin, 10% (v/v) heat-inactivated FBS, 100 U mL^−1^ penicillin and 100 μg mL^−1^ streptomycin, in a Haier Biomedical incubator (model HCP-168) at 37 °C in a humidified atmosphere containing 5% CO_2_. The culture medium was changed twice a week, and when 80% confluence was reached, the cells were detached with 0.25% trypsin/EDTA. Cells were cultured at a density of 5.0 × 10^5^ cells per mL in a 75 cm^2^ culture flask in the complete DMEM medium mentioned previously and were maintained at 37 °C with 5% CO_2_, and controlled humidity for 9 days to allow differentiation.

### Cell viability: MTT assay

The MTT assay, which relies on the reduction of MTT to formazan, was conducted to assess the metabolic activity of C2BBe1 in response to the treatment with the compounds under study. The assay was first optimised regarding the cell density, the seeding plate, the potential impact of medium aspiration on cell detachment, and the maximum concentration of DMSO compatible with the assay, with tested concentrations between 0.1 to 1% (v/v).

Following optimisation, the cells were seeded into 96-well plates at a concentration of 5 × 10^4^ cells per well in complete medium and incubated at 37 °C with 5% CO_2_ under a humidified atmosphere. After 9 days of differentiation, cells were treated with varying concentrations of myricetin (0–50 μM), genistein (0–50 μM), or acarbose (0–200 μM) dissolved in DMSO [final concentration of 0.25% (v/v)] for 10 minutes or 24 hours. At this time, the culture medium was replaced with a phenol red-free medium of the same composition. Following the exposure period, 10 μL of MTT solution (0.5 mg mL^−1^ in DPBS) was added to each well, and the cells were incubated for 2 hours at 37 °C with 5% CO_2_. Subsequently, the medium was removed, and the resulting purple formazan crystals were solubilised with 100 μL of DMSO per well. After 30 minutes of shaking at room temperature, the absorbance of the resulting solution, which reflects cellular metabolic activity, was measured using a microplate reader (Synergy HT, Bio-Tek) at 570 nm. The protocol was validated using Triton X-100 at a final concentration of 1% (v/v) as a positive control.

### Cell lysate preparation

After 9 days of differentiation, cell extracts were prepared according to the protocols of Geicu *et al.*^38^ and Ikeda *et al.*,^39^ with minor modifications. Cells were washed 3 times with cold DPBS and then scraped in 1 mL of 10 mM phosphate buffer (pH 7.0) containing protease inhibitors. Then, cells were disrupted by sonication (three times for 10 seconds with 20 seconds pause, on ice). After centrifugation at 10 000 g for 10 minutes at 4 °C, the supernatant was collected and stored at −20 °C until required.

### Determination of protein concentration

Protein concentration of cell lysate was determined using the Bio-Rad Protein Assay kit (Hercules, CA, USA) based on the Lowry method. According to the kit instructions, in a 96-well plate, 5 μL of cell extract was mixed with 25 μL of working reagent A′ - prepared by combining 1 mL of reagent A with 20 μL of reagent S - and 200 μL of reagent B. The samples were mixed thoroughly and incubated at room temperature for at least 15 minutes. Absorbance at 750 nm was then measured using a microplate reader (Synergy HT, Bio-Tek). BSA standards (0.25–2.0 mg mL^−1^), dissolved in the same solvent as the cell lysate (10 mM phosphate buffer, pH 7.0), were used to generate the standard curve, and the resulting equation was employed to calculate the protein content of the cell extract.

### α-Glucosidase inhibitory activity in C2BBe1 - *p*NPG assay

The assay was performed following the same principle described in the previous section. Prior optimisation of the enzymatic assay was performed by varying incubation time (0–120 minutes), total protein added (0–600 μg mL^−1^), and final DMSO concentration (0–1.0%). Based on these initial tests, the ideal experimental conditions for this assay were established.

The assay was carried out in a 96-well microplate, as described by Proença *et al.*,^21^ with some modifications. Briefly, cell extracts were diluted in 10 mM phosphate buffer (pH 7.0) to obtain 400 μg mL^−1^ of total protein per well. Samples were incubated at 37 °C for 10 minutes with the selected flavonoids (0–50 μM) or DMSO [final concentration was 0.25% (v/v)]. Then, a final concentration of 2.5 mM *p*NPG, prepared in 10 mM phosphate buffer (pH 7.0), was added. The enzymatic reaction was monitored for 2 hours using a microplate reader (Synergy HT, Bio-Tek), and the derivative values of the absorbance *versus* time plots in the interval 45 to 105 minutes were obtained and used for further calculation. Acarbose (0–200 μM) was used as a positive control.

### α-Glucosidase inhibitory activity in C2BBe1 – glucose oxidase assay

Since the *p*NPG assay commonly uses a synthetic substrate that may not accurately reflect the enzyme's natural activity, the glucose oxidase-peroxidase assay was developed, allowing the quantification of the amount of glucose released from the hydrolysis of natural substrates. However, polyphenols are known to interfere with this method due to their redox properties, including their ability to scavenge hydrogen peroxide, or through their inhibitory activity against glucose oxidase and/or peroxidase, the enzymes present in the kit.^36^ To determine possible interference with the Glucose Assay Kit, the selected flavonoids, at the maximum concentration (as evaluated by the MTT assay), were added to 300 μM glucose in the absence of the cell extract, in a 96-well microplate, following the kit manufacturer's instructions. A control sample (without any compound) was included for comparison. A decrease in the measured glucose levels only by the presence of the flavonoids would therefore indicate that the compounds interfere with one or more components of the kit. Therefore, only concentrations producing less than 20% inhibition of the glucose oxidase-peroxidase developing reaction were used in subsequent experiments. Acarbose, used as the positive control, did not interfere with the mentioned assay even at the highest concentration tested (100 μM). The assay was carried out in a 96-well microplate according to the procedure described by Proença *et al.*^21^ with minor modifications. Briefly, cell extracts corresponding to 400 μg mL^−1^ of the total protein were added to each well along with the tested flavonoid (0–50 μM) or DMSO [final concentration was 0.25% (v/v)]. After incubation at 37 °C for 10 minutes, maltose (final concentration of 1 mM in 10 mM phosphate buffer) was added. The reaction mixture was incubated for additional 45 minutes at 37 °C in a thermoshaker. Glucose released from substrate hydrolysis was then quantified using the Glucose Assay Kit, according to the manufacturer's instructions. After incubating at room temperature for 30 minutes, absorbance was measured at 570 nm using a microplate reader (Synergy HT, Bio-Tek).

### GC–MS analysis

Glucose generated during the enzymatic reaction was additionally measured by GC–MS. Briefly, 100 μL of cell extract corresponding to 400 μg mL^−1^ of the total protein was incubated with the tested flavonoid (0–50 μM) or DMSO [final concentration was 0.25% (v/v)] for 10 minutes at 37 °C. Subsequently, 100 μL of maltose (final concentration of 1 mM in 10 mM phosphate buffer) was added, and the reaction was allowed to proceed for an additional 45 minutes at 37 °C. The enzymatic reaction was then terminated by freezing, followed by lyophilisation for 24 hours. The dried samples were stored at −80 °C until required.

Sample preparation and chromatographic analysis were performed according to the established protocol of Amaro *et al.*^40^ On the day of GC–MS analysis, 10 μL of the internal standard (desmosterol 1 mg mL^−1^ in chloroform) was added to all samples, then dried under a nitrogen stream. For derivatisation, 50 μL of MOX (20 mg mL^−1^ in pyridine) was added to the dried extracts, followed by incubation at 60 °C for 60 minutes. Then, 100 μL of BSTFA+TMCS was added, and the samples were incubated for 60 minutes at 60 °C. For chromatographic analysis, 2 μL were injected into the GC–MS in split mode (1/20).

The analysis was performed using a Bruker 436-GC model coupled to a SCION single quadrupole (SQ) mass spectrometer (Bruker Daltonics), and a Combi-PAL autosampler (Varian), and Bruker MS Workstation software (version 8.2.1, Bruker Daltonics, Bremen, Germany). Separation was achieved using an Rxi-5Sil MS column (30 m × 0.25 mm × 0.25 μm, Restek Corporation, U.S., Bellefonte, PA, USA) with helium carrier gas (C-60, Gasin, Porto, Portugal) at a constant flow rate of 1 mL min^−1^. The oven temperature program was as follows: initial hold at 70 °C for 2 minutes, ramped to 250 °C at 15 °C min^−1^ (2 minutes hold), then ramped to 300 °C at 10 °C min^−1^ (5 minutes hold). The injector temperature was maintained at 250 °C, with transfer line, ion source, and manifold temperatures set to 250 °C, 260 °C, and 39 °C, respectively. Electron ionisation (EI) was performed at 70 eV in full-scan mode, acquiring data across a mass-to-charge (*m*/*z*) range of 50–1000.

The data (retention time and peak area) of both glucose and maltose were exported and normalised to area of the internal standard (desmosterol). Then, the percentage inhibition was determined by the following formula:
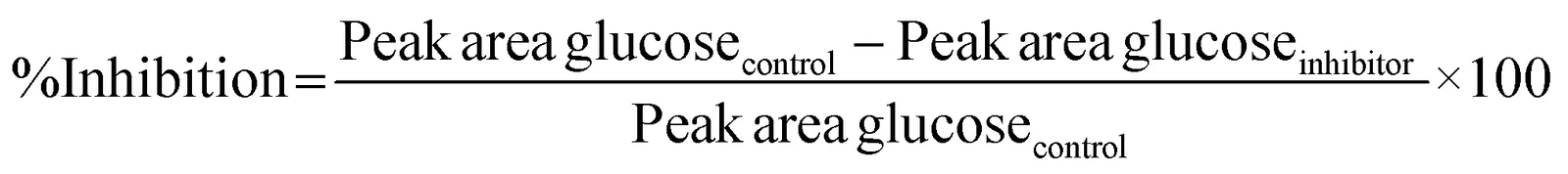


### Statistical analysis

The results of the *in vitro* inhibitory activities of the compounds against both pancreatic α-amylase and α-glucosidase are expressed as mean ± standard error of mean (SEM) and represent at least three independent experiments. A statistical comparison of the active compounds was performed by applying the one-way analysis of variance (ANOVA). Differences were considered to be significant at *p-*values lower than 0.05. All the statistical analyses were performed using GraphPad Prism™ (version 9.0; GraphPad Software).

Lineweaver–Burk plots were analysed for each inhibitor and substrate concentration, and the kinetic parameters used to determine the inhibition type were calculated using GraphPad Software 9.0.

### Conclusions

Initial screening using α-glucosidase identified seven active compounds, all displaying greater inhibitory potency than acarbose. Among them, myricetin and genistein emerged as the most potent and exhibited no or substantially lower inhibitory effects on α-amylase.

When the human enzyme was evaluated using the physiological substrate, maltose, myricetin produced nearly 30% of inhibition at 6.25 μM, whereas genistein showed no inhibitory activity at the highest concentration tested (50 μM). To enable the evaluation of higher concentrations of myricetin, GC–MS was employed as an alternative analytical approach. This method confirmed myricetin's inhibitory activity against human α-glucosidase, with more than 50% of enzyme inhibition achieved at 25 μM.

Notably, myricetin was the most active flavonoid evaluated, suggesting that the presence of –OH groups at position 3 of the C ring, at positions 3′, 4′ and 5′ of the B ring, and at positions 5 and 7 of the A ring contribute to enhanced α-glucosidase inhibitory activity.

Overall, these findings support further investigation of these compounds as potential modulators of additional pathways involved in the pathogenesis of diabetes and its associated complications.

## Author contributions

Conceptualization: M. Luísa Corvo, Eduarda Fernandes, and Marisa Freitas; methodology: Beatriz Vicente, Filipa Amaro, and Paula Guedes de Pinho; investigation: Beatriz Vicente, Filipa Amaro, Paula Guedes de Pinho, and Carina Proença; writing – original draft preparation: Beatriz Vicente; writing – review and editing: Filipa Amaro, Paula Guedes de Pinho, Carina Proença, M. Luísa Corvo, Eduarda Fernandes, and Marisa Freitas; funding acquisition: M. Luísa Corvo, Eduarda Fernandes, and Marisa Freitas; supervision: M. Luísa Corvo, Eduarda Fernandes, and Marisa Freitas. All authors have read and agreed to the published version of the manuscript.

## Conflicts of interest

There are no conflicts to declare.

## Supplementary Material

MD-017-D6MD00685J-s001

## Data Availability

The data supporting this article have been included as part of the supplementary information (SI). Supplementary information is available. See DOI: https://doi.org/10.1039/d6md00685j.
