# Supplementary material for: Flavonoids and chalcones as selective α-glucosidase inhibitors: from enzymatic screening to validation using the human enzyme
Source: RSC Med Chem. 2026 Sep 10;17(10):4838–50. doi: 10.1039/d6md00685j (PMC13559892; doi:10.1039/d6md00685j)
Supplement: Supplementary file 1 [file MD-017-D6MD00685J-s001.pdf]

Supplementary Information (SI) for RSC Medicinal Chemistry.
This journal is © The Royal Society of Chemistry 2026
Supplementary Information (SI) for RSC Medicinal Chemistry.
This journal is © The Royal Society of Chemistry

Flavonoids and chalcones as selective α-glucosidase inhibitors: from enzymatic screening to validation using the human enzyme

Beatriz Vicente[a], Filipa Amaro[b,c], Paula Guedes de Pinho[b,c], Carina Proença[a], M. Luísa Corvo[d], Eduarda Fernandes*[a], Marisa Freitas*[a]

[a] *LAQV, REQUIMTE, Laboratory of Applied Chemistry, Department of Chemical Sciences, Faculty of Pharmacy, University of Porto, Porto, Portugal. E-mail:egracas@ff.up.pt; marisafreitas@ff.up.pt.*

[b] *Associate Laboratory; i4HB–Institute for Health and Bioeconomy, University of Porto, 4050-346 Porto, Portugal.*

[c] *UCIBIO–Applied Molecular Biosciences Unit, Laboratory of Toxicology, Faculty of Pharmacy, University of Porto, 4050-346 Porto, Portugal.*

[d] *Research Institute for Medicines, Faculdade de Farmácia, Universidade de Lisboa, 1649-003 Lisbon, Portugal.*

Supplementary information

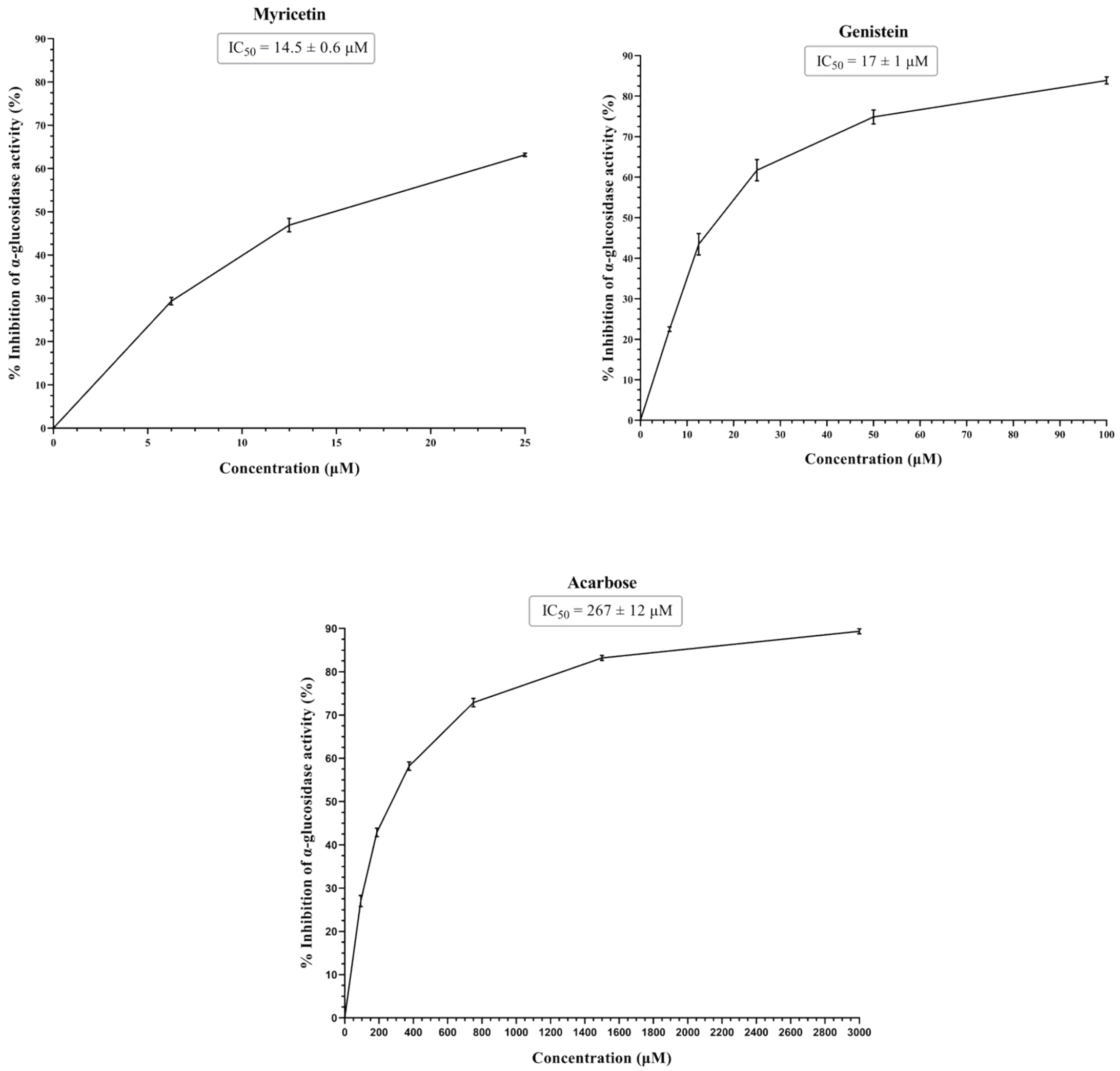

Supplementary Figure 1 α-Glucosidase inhibition by myricetin, genistein and acarbose, the positive control. Each value represents mean ± SEM of at least three experiments.
